# Low interstitial fluid in patients with spontaneous intracranial hypotension

**DOI:** 10.1186/s12987-026-00785-7

**Published:** 2026-03-03

**Authors:** Charlotte Zander, Alexander Rau, Niklas Lützen, Katharina Wolf, Florian Volz, Amir El Rahal, Laura Krismer, Hansjörg Mast, Marco Reisert, Elias Kellner, Jürgen Beck, Horst Urbach, Theo Demerath

**Affiliations:** 1 Department of Neuroradiology, Medical Center, Faculty of Medicine, University of Freiburg, Freiburg, Germany; 2 Department of Neurosurgery, Medical Center, Faculty of Medicine, University of Freiburg, Freiburg, Germany; 3 Department of Radiology, Medical Center, Faculty of Medicine, University of Freiburg, Freiburg, Germany

**Keywords:** Neuroimaging, Cerebrospinal fluid loss, Cerebrospinal fluid disorder, Advanced imaging, Glymphatics

## Abstract

**Background:**

CSF loss in spontaneous intracranial hypotension (SIH) has been related to alterations in glymphatic flow, which is poorly understood in this disease. Advanced multi-shell diffusion-weighted MRI (dMRI) enables quantification of the interstitial free water fraction, serving as a possible surrogate marker for glymphatic system function in patients with SIH.

**Methods:**

SIH Patients underwent dMRI before and after closure of a spinal CSF leak. The microstructural free water compartment (V-CSF) of the whole brain gray and white matter, corona radiata, amygdala, hippocampi and parahippocampal gyri was compared to 23 age-matched normal controls. Pre- and post-therapeutic volumetry encompassed the total ventricular, total gray and white matter compartments and mesial temporal structures.

**Results:**

23 SIH patients (50.3 ± 13.1 years, 15 women) were included. After leak closure, V-CSF increased in the global gray matter (mean pre 0.140 vs. mean post 0.151; *p* = 0.029), posterior corona radiata (mean pre 0.103 vs. mean post 0.108; *p* = 0.0055), hippocampi (mean pre 0.100 vs. mean post 0.105; *p* = 0.001), and parahippocampal gyri (mean pre 0.156 vs. mean post 0.177; *p* = 0.009). Compared to normal controls, V-CSF was decreased before leak closure in the hippocampi (mean pre 0.100 vs. mean NC 0.211; *p* = 0.0019) and posterior corona radiata (mean pre 0.103 vs. mean NC 0.118; *p* = 0.011). No significant change of total gray or white matter volume occurred after leak closure.

**Conclusion:**

Closure of the spinal CSF leak leads to an increase of interstitial fluid in gray matter, corona radiata, hippocampi, and parahippocampal gyri, respectively. Our results suggest, that SIH patients may have less interstitial fluid in the hippocampi and posterior corona radiata compared to normal controls. Whether shifts in brain interstitial fluid in eloquent cerebral regions contribute to cognitive decline in patients with CSF loss should be topic of further research.

## Introduction

Spontaneous intracranial hypotension (SIH) is a debilitating disease resulting from spinal CSF loss [1]. The Monro-Kellie doctrine states that the volume loss of one intracranial compartment (i.e. brain, blood, CSF) leads to an increase of the others to maintain an equilibrium [2]. Regarding SIH, recent studies quantified macroscopic shifts in these three components before and after leak closure. Volumetric shifts comprised the CSF and venous compartments, whereas there was no significant change in the total gray or white matter compartments [3, 4]. 

In addition to such a macroscopic perspective, biophysically motivated MRI techniques relying on multishell diffusion-weighted MRI sequences such as Diffusion Microstructure Imaging (DMI), enable the in vivo investigation of the cerebral microcompartments, i.e. intra-axonal, extra-axonal and free water CSF compartment [5]. This methodology has been successfully applied in epilepsy, neurodegenerative, neurooncological, and infectious diseases, providing deeper insights into underlying microstructural changes [6–9]. For instance, this technique facilitated correlating distinct symptoms of the post-COVID syndrome with specific brain regions [6]. 

Typical symptoms in SIH encompass orthostatic headache, neck stiffness and hearing disturbances; however, spinal CSF loss can also result in more serious complications, such as subdural hematoma or even coma [10–12]. While cognitive decline in patients with SIH is increasingly recognized and was shown to attenuate after successful therapy, there is still a lack in understanding the underlying pathophysiological mechanisms [13, 14]. The manifestations of those cognitive alterations vary from mild cognitive impairment to so-called brain-sagging or spinal dementia, which presents with symptoms comparable to behavioral variant of fronto-temporal dementia [13–15]. As a possible explanation, obstruction of the venous outflow as a consequence of the brain sagging has been discussed [15]. Furthermore, a recent study by Schievink et al. [16] identified azygos vein stenosis in patients with brain sagging dementia leading to a higher CSF to vein pressure gradient in 3/21 patients as a target for treatment. As shown by previous work, SIH-patients with mild cognitive impairment can improve after closure of a CSF-leak both regarding the Bern SIH-Score and also the MoCA Score, indicating a link between CSF homeostasis and cognitive deficits [14]. While different neurocognitive symptoms are typically associated with dysfunction in distinct brain regions, to date, the specific relationships between regional brain alterations and neurocognitive deficits have not been systematically investigated in patients with spontaneous intracranial hypotension.

At the meso-/microscopic level, the glymphatic system also appears to play a special role in understanding cerebral alterations: Current studies are discussing a possible influence of decreased glymphatic flow on neurocognitive changes. A recent study by Urbach et al. [17] provided indications of a reduced glymphatic flow in SIH. Another recent case series using diffusion tensor imaging along the perivascular space described increase in glymphatic flow, correlating with symptomatic improvement after leak closure [18]. In this context, changes in free interstitial fluid could serve as an indirect indicator for glymphatic flow.

Given that CSF loss represents the central pathomechanism in SIH, analyzing regional fluid shifts – not merely between ventricular and parenchymal spaces but specifically across microstructural compartments – is of particular interest. We hypothesize that patients with SIH have an altered relative free water distribution in the brain tissue which is modified by the closure of the spinal CSF leak.

## Materials and methods

This retrospective study was approved by the local Institutional Review Board (EK-Freiburg 24-1296-S1-retro). All procedures followed the ethical standards of the institutional and national research committee and comply with the 1964 Helsinki Declaration and its later amendments. Written informed consent was obtained from all participants. For inclusion, we screened consecutive patients with SIH diagnosed according to the International Classification of Headache Disorders [19] and referred to our institution between 10/2022 and 10/2023.

Inclusion criteria were as follows: (1) clinical suspicion for SIH with evidence for CSF loss in cranial and/or spinal MR imaging; (2) dynamic digital subtraction (DSM) or CT myelography (CTM) demonstrating the level of the spinal CSF leak; (3) targeted therapy through surgical leak closure, transvenous embolization or fibrin patching; (4) cranial MR imaging with high-resolution isotropic T1-weighted and multishell diffusion-weighted MRI sequences before and after therapy. Patients with poor-quality MR imaging or subdural hematoma were excluded due to the specific limitations and requirements of the chosen methodological approach. Our study included an age-matched cohort of normal controls (NC).

### Clinical data

Clinical data at baseline encompassed the patients’ age, sex, and duration of symptoms. Leak types according to the result of the myelography were reported. Headache intensity was rated at baseline and 3-month follow-up in 20/23 patients using the six-item Headache Impact Test (HIT-6™) [20] with a total score between 36 and 78, quantifying the impact of headaches on daily life.

For neurocognitive testing, the Montreal Cognitive Assessment (MoCA© version 8.1 and 8.2) [21] was performed at baseline before any invasive diagnostics and repeated 36–72 h after treatment. A maximum score of 30 points can be achieved on this test, with a score of 26–30 points indicating normal cognitive performance. Results were available in 9/23 patients before and 7/23 patients after treatment. These were already published in a prior investigation [14]. 

### Cerebral MR imaging

Pre- and post-treatment MRIs were performed on 3T scanners (Magnetom Prisma/Prisma fit; Siemens Healthineers, Erlangen, Germany) using 64 channel head coils. A pre-contrast isotropic (1mm^3^) T1w 3D magnetization-prepared 180° radio‐frequency pulse and a rapid gradient‐echo (MP‐RAGE) sequence (Prisma/Prisma fit: repetition time 2500 ms; echo time 2.82 ms; flip angle 7°, TI 1100 ms; GRAPPA factor = 2; 1.0 mm isotropic voxels, 176 contiguous sagittal slices) and a multi-shell diffusion imaging sequence were acquired using the following parameters: 42 slices in axial orientation, voxel size 1.5 × 1.5 × 3 mm³, TR 2800 ms, TE 88 ms, bandwidth 1778 Hz, flip angle 90°, simultaneous multi-band acceleration factor 2, GRAPPA factor 2, 15 non-diffusion weighted images, 2 × 58 images with b-factors 1000 and 2000 s/mm^2^; 17 diffusion directions for b-factor 0 and 58 diffusion directions each for b-factors 1000 and 2000 s/mm^2^. MRI was acquired at mean 10.1 (± 40.1) days prior to treatment. Post-treatment brain MRI was performed at a mean of 8.6 (± 9.9) days after leak closure.

Data of normal controls were obtained from an institutional normal collective database (EK-Freiburg 104/20).

### Imaging evaluation

Brain MRIs were analyzed for evidence of SIH at baseline and follow-up using the Bern SIH score [22]. The total score was used to classify a patient’s probability for finding a spinal CSF leak: ≤2 = low, 3–4 = intermediate, and ≥ 5 = high. Pachymeningeal enhancement and venous engorgement were assessed using 3D-FLAIR, adapted from a study by O’Cearbhaill et al. [23].

### Image postprocessing

Image postprocessing was conducted using a local instance of the NORA processing platform (www.nora-imaging.org). T1w images were segmented into white matter (WM), gray matter (GM), and CSF with SPM12 (Wellcome Centre for Human Neuroimaging, London, UK) [24]. 

**Fig. 1:**
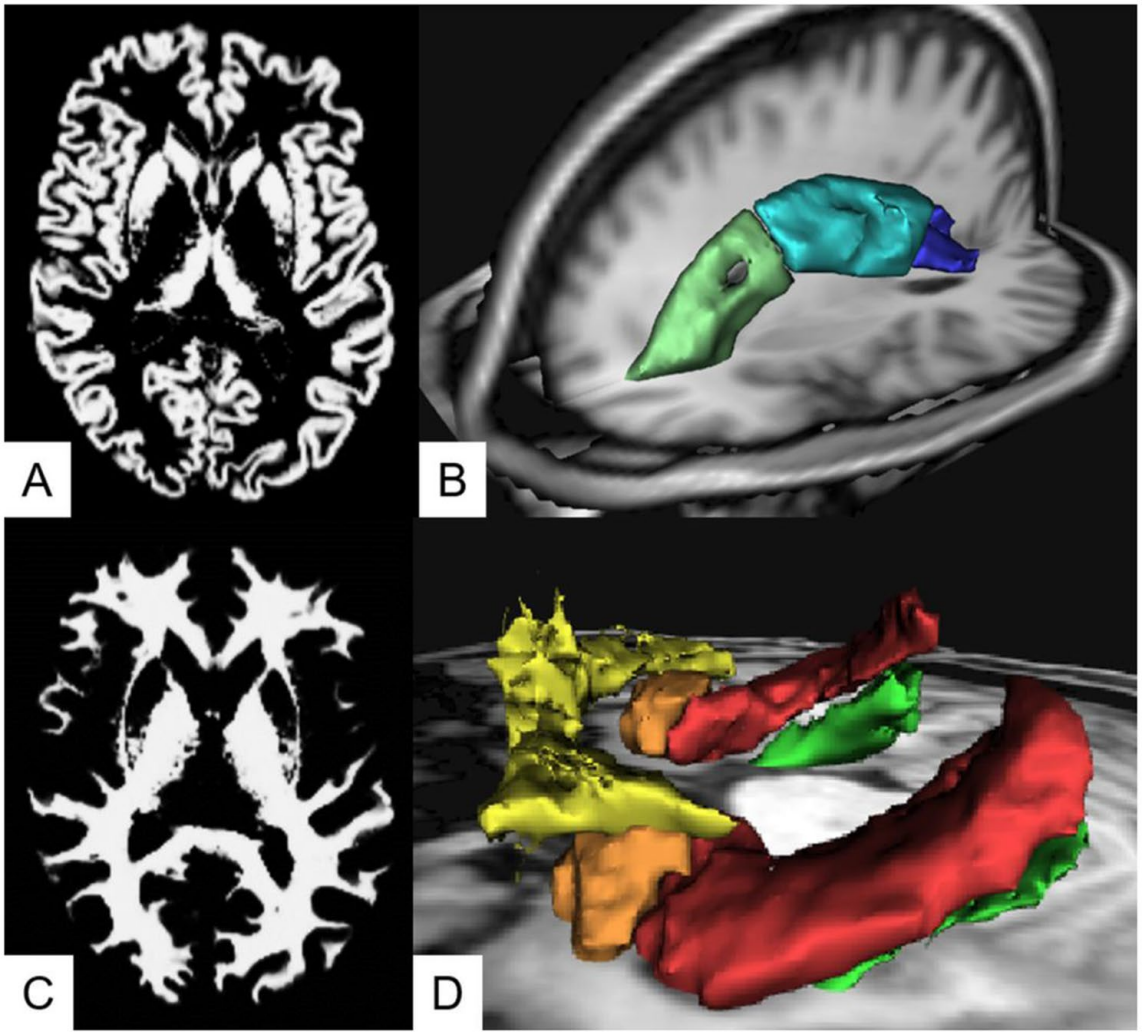
Segmentation techniques. (**A**) SPM12-based gray matter segmentation. (**B**) Atlas based segmentations of the anterior (green), superior (light blue), and posterior (dark blue) corona radiata. (**C**) SPM12-based white matter segmentation. (**D**) Freesurfer- and Atlas-based segmentations of mesial temporal structures (yellow: orbitofrontal cortex, orange: amygdala, red: hippocampus, green: parahippocampal gyrus)

The total gray and white matter volumes and total intracranial intraventricular volumes were obtained from SPM12 (Fig. 1) [4, 24]. In order to avoid spatial brain shift effects on our measurements, which inevitably occur due to treatment and subsequent brain shift, we used individual FreeSurfer-based segmentations of the hippocampus, amygdala, and parahippocampal gyri (FreeSurfer V6.0 toolbox with default parameters, parcellation was performed using the Desikan-Killiany atlas) [25, 26]. Regarding diffusion imaging postprocessing, as a first pre-processing step, denoising [27] and Gibbs-artifact removal [28] was performed. DMI metrics were calculated using a Bayesian approach [5]. In the present work, we focus on the “free-fluid” (V-CSF) space of a commonly used three compartment model [5]. 

### Spinal imaging

All patients underwent unenhanced heavily T2-weighted fat-saturated whole spine MR imaging on admission. Based on the results of this, DSM and CTM were performed as previously described [29, 30]. DSM was performed with the patient in the prone position if a ventral leak was suspected on spinal MR imaging, or in a lateral decubitus position if a lateral leak was suspected. 13 patients were suspected to have a CVF, as spine MRI was negative for a spinal longitudinal extradural CSF collection. All of these underwent CTM (+/- DSM), confirming the presence of a CVF in six patients and a CSF-lymphatic fistula in one patient [30, 31]. 

### Targeted therapy

All surgeries were performed via a minimally invasive tubular approach as recently described [32–35]. 

Transvenous embolization using Onyx was conducted via a femoral access with venous access via the azygos or hemiazygos/accessory hemiazygos vein as previously described [36]. A follow-up CT was obtained to confirm the correct level.

Fibrin patching was adapted from a study by Mamlouk et al. [37] using 2–4 ml fibrin glue (Tisseel Prima 4 mL; Baxter). The outcome was verified by a follow-up MRI, showing total resolution of the extradural fluid.

### Statistical analysis

The assumption of normal data distribution was tested with Shapiro-Wilk test. Descriptive analysis was performed using frequencies and percentages for categoric variables and mean (+/- SD) or median (interquartile range) for continuous variables. Differences between pre- and post-treatment metrics were assessed with paired t-tests and Wilcoxon sided rank tests as appropriate. An α-level of 0.05 was considered statistically significant. All statistical analyses were performed using GraphPad Prism (Version 10.2.3).

## Results

### Study population

Between August 2022 and December 2023, a total of 23 SIH patients who met inclusion criteria were identified and treated in our center. 15 patients were female (65%), and the mean age was 50.3 ± 13.1 years (range 31.3–69.5 years). Median symptom duration was 282 days (IQR 114–658). Mean SIH score in initial MRI of the head was 5.3 ± 2.6 (range 2–9), decreasing to 2.4 ± 2.1 (range 0–7, *p* < 0.0001) at post-treatment imaging. Patient characteristics are presented in Table 1.

16 patients had extradural fluid collections on spinal MRI. Of these, 14 had a ventral dural tear (Type I leak) and two a lateral dural tear (Type II leak). Six patients had a CVF, of whom one presented with two fistulas at different levels. Another patient had a primary CSF lymphatic fistula [31]. 21 patients underwent open surgery, one patient with CVF underwent transvenous embolization with an ethylene-vinyl alcohol based agent, and another patient with type 2 leak received CT-guided fibrin patching.

HIT scores were available in 20/23 patients. There was a significant clinical improvement with a median HIT score of 66.0 (IQR 63.25–68.75) before and 48.5 (IQR 42.0–54.0) after therapy (*p* < 0.0001). MoCA score was available in 9/23 patients before and 7/23 patients after treatment. There was an increase in all 7/7 patients with follow-up data with a median MoCA before treatment of 27 (IQR 24.5–28.5) and a median MoCA after of 28 (IQR 28.0–29.0).

**Table 1:** Patient characteristics

No.	Sex	Age (years)	Duration of Symptoms (days)	Bern SIH Score Pre	Bern SIH Score Post	Leak site	Leak Type^a^	Treatment	HIT-6 Pre	HIT-6 Post
**1**	F	35	3401	2	1	T 10/11 left	2	FP	50	N/A
**2**	M	36	289	8	3	T 2/3 ventral	1	OS	66	53
**3**	F	62	843	9	6	T 8/9 ventral	1	OS	47	45
**4**	M	60	178	5	3	T 9/10 right	3	OS	66	50
**5**	F	36	132	7	1	T 2/3 ventral	1	OS	60	46
**6**	F	48	114	2	1	T12/L1 right	2	OS	67	62
**7**	F	61	287	5	0	T 10/11 ventral	1	OS	71	55
**8**	M	59	434	3	4	T 9/10, T10/11 right	3	EMB	68	48
**9**	M	69	5374	2	2	T 2/3 ventral	1	OS	N/A	N/A
**10**	F	65	140	8	3	T 9/10 right	3	OS	63	54
**11**	F	42	71	3	0	T 7/8 ventral	1	OS	63	36
**12**	F	60	62	7	1	T 8/9 ventral	1	OS	70	38
**13**	F	37	282	6	6	T 10/11 ventral	1	OS	64	54
**14**	F	35	354	4	3	T 3/4 ventral	1	OS	65	50
**15**	F	61	2720	7	7	T 6/7 right	3	OS	68	40
**16**	F	33	2055	4	4	T 1/2 ventral	1	OS	73	42
**17**	M	57	263	9	1	T 6/7 right	3	OS	76	58
**18**	M	38	44	9	5	T 2/3 ventral	1	OS	69	42
**19**	F	65	115	2	0	T 7/8 left	3	OS	65	46
**20**	M	42	375	2	0	T 11/12 ventral	1	OS	59	57
**21**	F	59	84	8	0	T 3/4 ventral	1	OS	66	49
**22**	F	31	658	4	3	L 4/5 left	CLF	OS	67	40
**23**	M	65	84	7	2	C7/T1 ventral	1	OS	64	N/A

No.: Patient number; SIH: Spontaneous intracranial hypotension; Pre: Pre-therapeutic; Post: Post-therapeutic; HIT-6: Headache impact Test; CLF: CSF-lymphatic fistula; FP: CT-guided fibrine patch; OS: open surgery; EMB: transvenous embolization. ^a^Leak types were classified according to Schievink et al. [1]

### Longitudinal free water distribution within the gray and white matter in SIH patients before and after leak closure

**Fig. 2:**
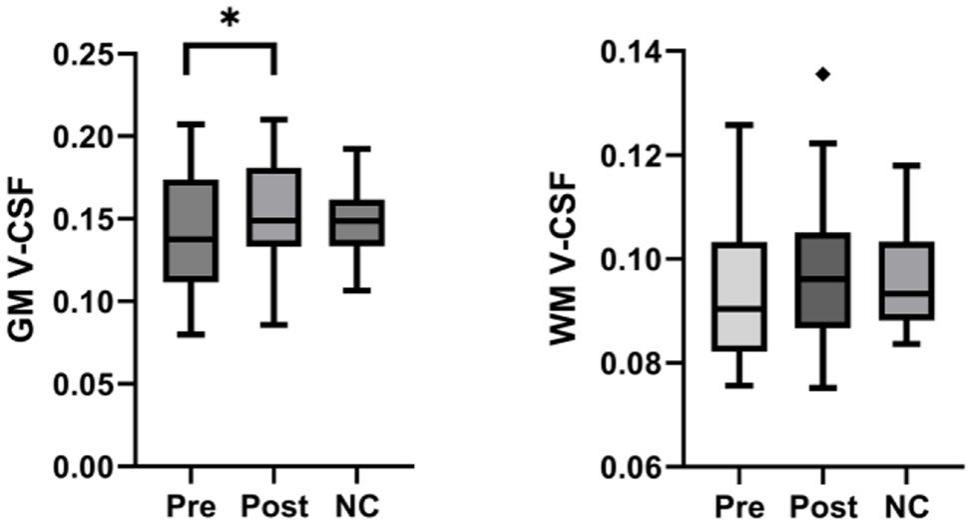
Free-water fraction of the gray and white matter. Boxplots of regional free-water fraction (V-CSF) in 23 spontaneous intracranial hypotension (SIH) patients before (Pre) and after (Post) therapy versus 23 age-matched normal controls (NC) show a significant increase in gray matter (GM) V-CSF and a positive trend in white matter (WM) V-CSF after spinal leak closure. * *p* < 0,05

Comparing paired data in SIH patients before and after leak closure, we observed a significant increase in gray matter V-CSF (mean pre 0.140, 95%CI 0.124–0.156 vs. mean post 0.151, 95%CI 0.136–0.166; *p* = 0.029), and higher values without statistical significance in white matter V-CSF (mean pre 0.094, 95%CI 0.088-0.100 vs. mean post 0.097, 95%CI 0.088-0.100; *p* = 0.182). There was, however neither before nor after therapy a significant difference of the gray or white matter V-CSF compared to age-matched normal controls (Figs. 2 and 5).

**Fig. 3:**
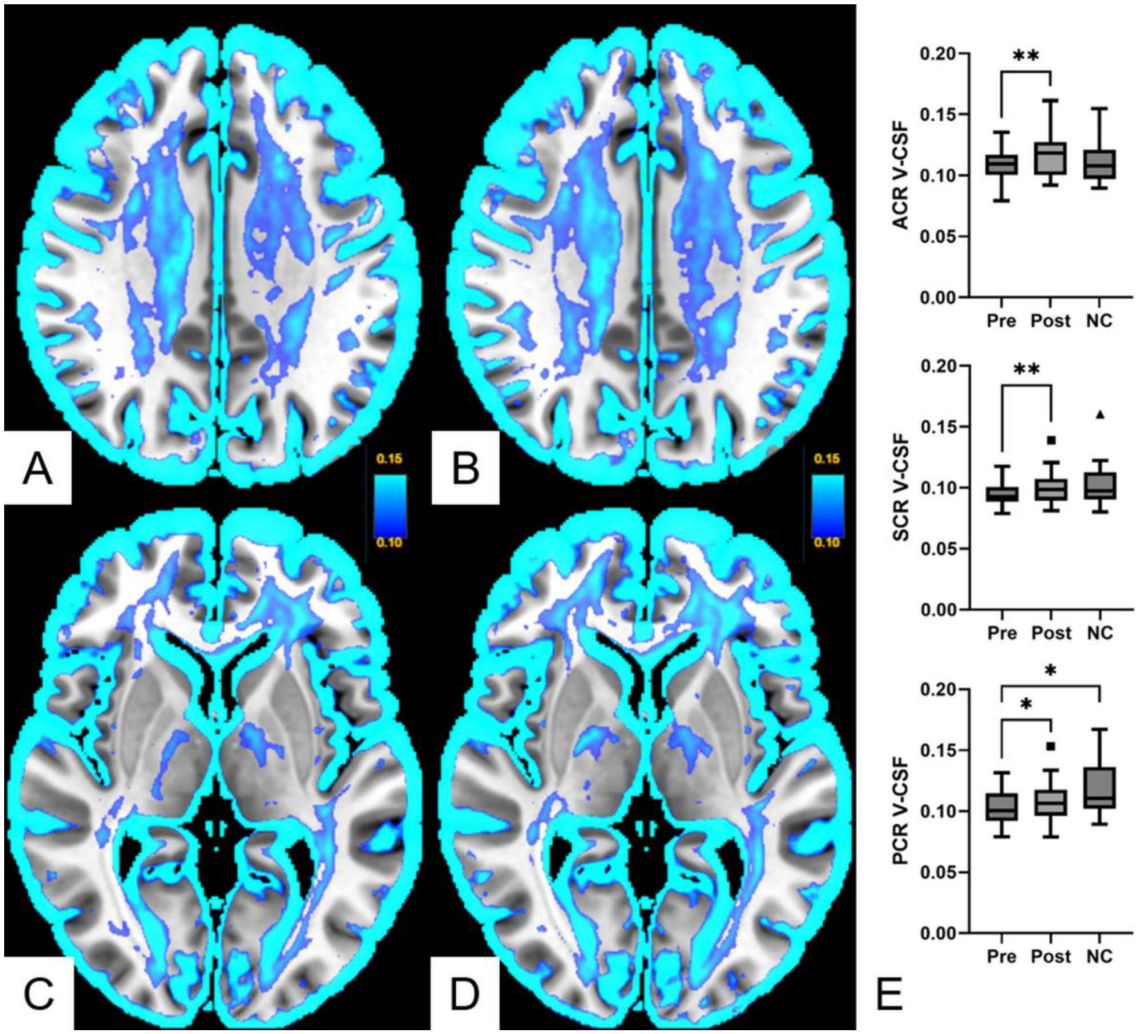
Free-water fraction within the corona radiata. Mean free interstitial water (V-CSF) maps of 23 spontaneous intracranial hypotension (SIH) patients before (**A**, **C**: Pre) and after (**B**, **D**: Post) closure of a CSF leak. There was a significant increase in V-CSF in the anterior (ACR, **E**), superior (SCR, **E**), and posterior (PCR, **E**) corona radiata. Compared to 23 age-matched normal controls (NC), V-CSF in the posterior corona radiata was significantly lower (**E**). * *p* < 0,05; ** *p* < 0,01

We separated the anterior, superior and posterior corona radiata using the WMPM Type III brain atlas [38]. There was a significant increase in V-CSF in the anterior (mean pre 0.109, 95%CI 0.102–0.116 vs. mean post 0.116, 95%CI 0.109–0.124; *p* = 0.005), superior (mean pre 0.094, 95%CI 0.090–0.099 vs. mean post 0.099, 95%CI 0.094–0.105; *p* = 0.0066), and also posterior corona radiata (mean pre 0.103, 95%CI 0.097–0.109 vs. mean post 0.108, 95%CI 0.101–0.116; *p* = 0.0055). There was no significant difference between pre- or post-treatment V-CSF and normal controls measures in the anterior and superior corona radiata. However, compared to normal controls, we found a significant decrease in pre-therapeutic V-CSF in the posterior corona radiata (mean pre 0.103, 95%CI 0.097–0.109 vs. mean NC 0.118, 95%CI 0.108–0.128; *p* = 0.011), see Figs. 3 and 5.

### Longitudinal free water distribution within the basal forebrain and mesiotemporal structures in SIH patients before and after leak closure

We found a significant increase of V-CSF in the hippocampi after treatment (mean pre 0.100, 95%CI 0.088–0.113 vs. mean post 0.105, 95%CI 0.093–0.116; *p* = 0.001), which was also present in the parahippocampal gyri (mean pre 0.156, 95%CI 0.136–0.176 vs. mean post 0.177, 95%CI 0.150–0.203; *p* = 0.009). There also was a distinct positive trend to increased V-CSF in the amygdala (mean pre 0.100, 95%CI 0.088–0.113 vs. mean post 0.105, 95%CI 0.093–0.116; *p* = 0.339) after treatment.

**Fig. 4:**
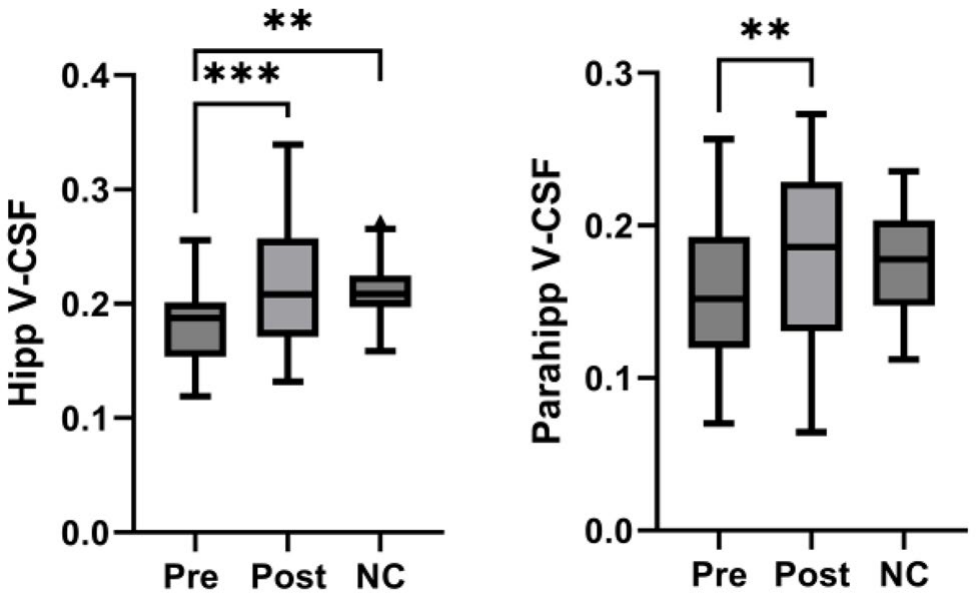
Mesiotemporal free-water fraction. Free-water fraction (V-CSF) in the hippocampi (Hipp) and parahippocampal gyri (Parahipp) significantly increased after (Post) compared to before (Pre) treatment in 23 patients with spontaneous intracranial hypotension (SIH). Compared to 23 age-matched normal controls (NC), there was a significant decrease in hippocampal V-CSF before treatment. ** *p* < 0,01; *** *p* < 0,001

**Fig. 5:**
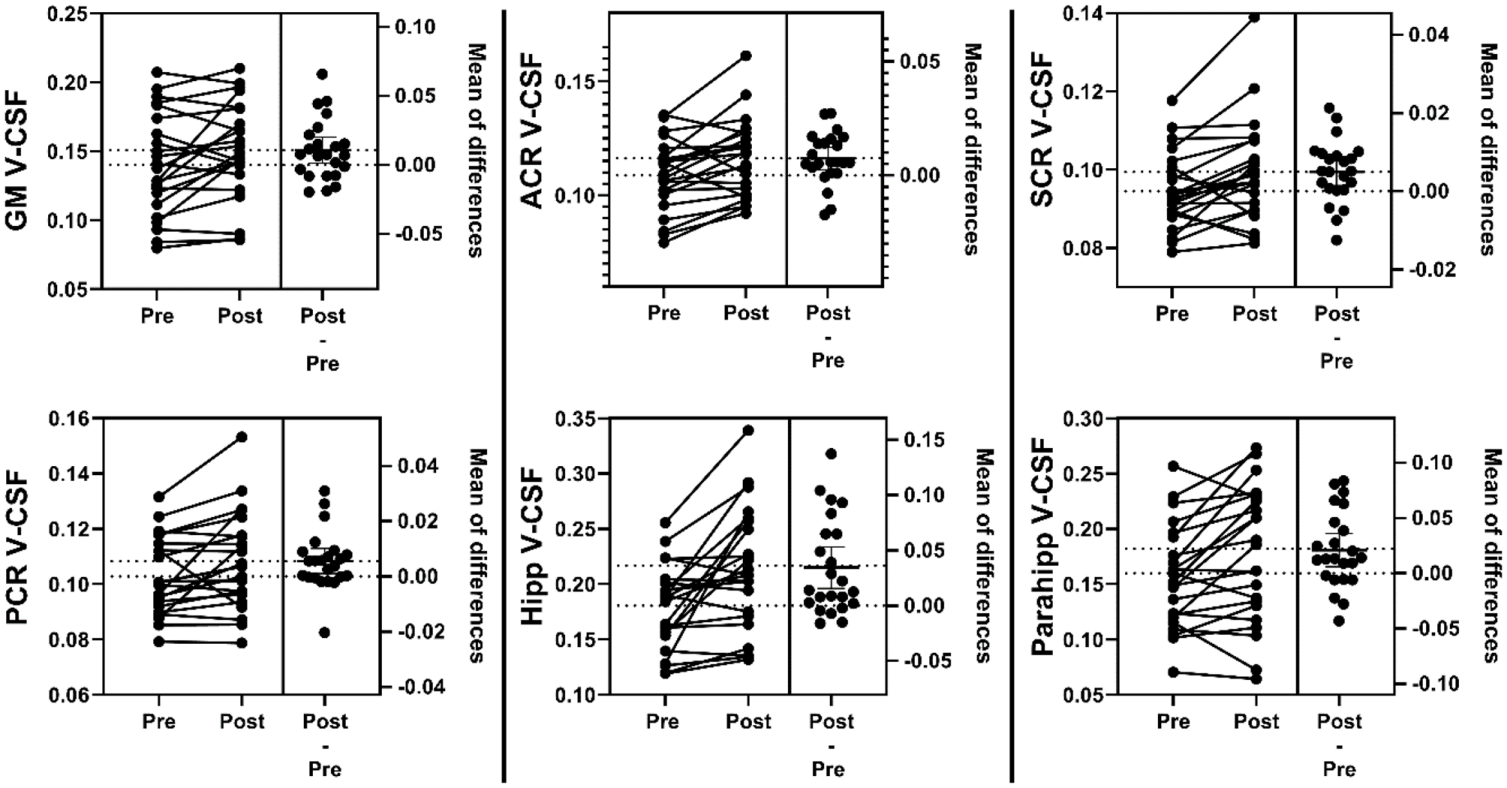
Tissue free-water fraction. Estimation plots of paired free-water fraction (V-CSF) values in 23 spontaneous intracranial hypotension (SIH) patients before (Pre) and after (Post) therapy. Values represent mean V-CSF of the total gray matter (GM), anterior/superior/posterior corona radiata (ACR, SCR, PCR), hippocampi (Hipp) and parahippocampal gyri (Parahipp)

In the hippocampi, we also found a significant decrease in V-CSF in SIH patients before treatment compared to normal controls (mean pre 0.100, 95%CI 0.088–0.113 vs. mean NC 0.211, 95%CI 0.199–0.224; *p* = 0.0019). There also was a slight trend to lower pre-treatment V-CSF in the parahippocampal gyri (mean pre 0.156, 95%CI 0.136–0.176 vs. mean NC 0.174, 95%CI 0.160–0.189; *p* = 0.13). Regarding the basal forebrain, there was no significant shift in V-CSF after leak closure (mean pre 0.159, 95%CI 0.142–0.177 vs. mean post 0.167, 95%CI 0.151–0.183; *p* = 0.192; Figs. 4 and 5).

### Total ventricular, gray matter, white matter, and mesial temporal volumes before and after closure of the CSF leak

Taking into account a water shift within the brain tissue, we also added volumetry of the target structures including the ventricles. On a global level, there was a significant increase in total ventricular volume (mean pre 213.1 ml, 95%CI 168.9-257.2 vs. mean post 242.2, 95%CI 194.8-289.6; *p* = 0.0012). We found no significant change in total gray matter (mean pre 310.5 ml, 95%CI 295.0-325.9 vs. mean post 306.7, 95%CI 292.5-320.9; *p* = 0.296) or total white matter volumes (mean pre 184.4 ml, 95%CI 173.3-195.4 vs. mean post 187.7, 95%CI 176.3-197.8; *p* = 0.091).

**Fig. 6:**
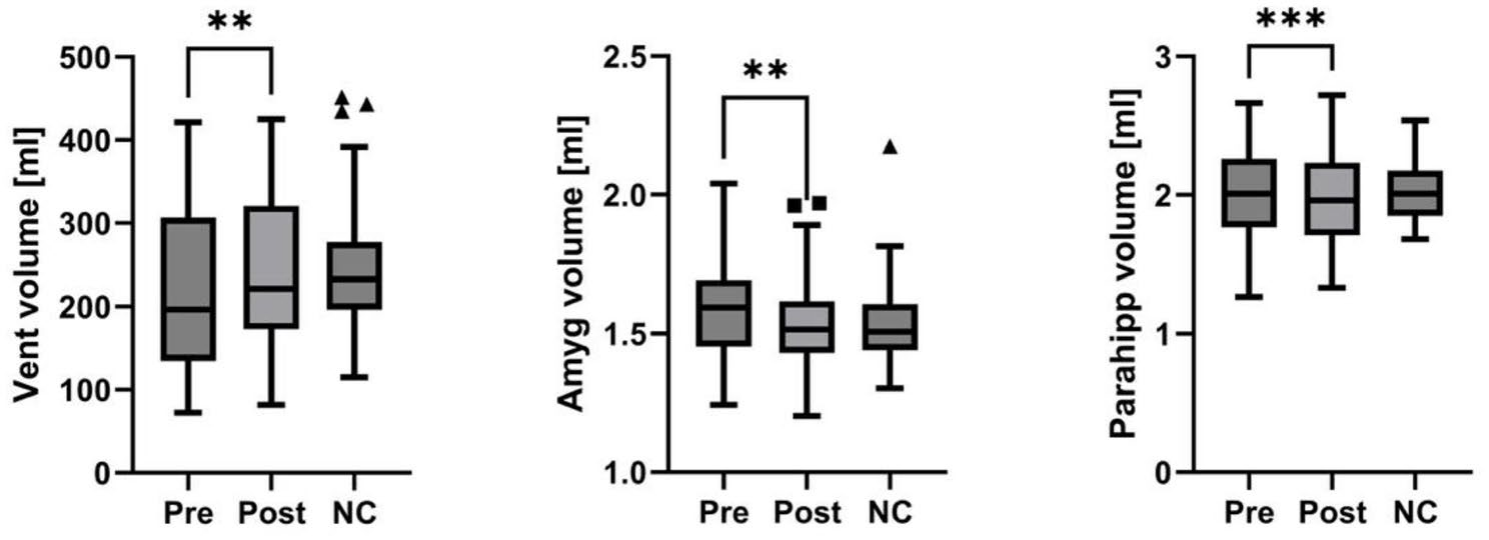
Total volumetric changes in patients with spontaneous intracranial hypotension before and after therapy vs. controls. Total ventricular (Vent), amygdala (Amyg), and parahippocampal (Parahipp) volumes of 23 patients with spontaneous intracranial hypotension (SIH) before (Pre) and after (Post) therapy compared with 23 age-matched normal controls (NC). Group comparisons revealed a significant increase in total ventricular volume post therapy, while there was a significant decrease in total volume of amygdala and parahippocampal gyri. ** *p* < 0,01; *** *p* < 0,001

Regarding the mesial temporal region, after leak closure we found a significant total volume decrease in the amygdala (mean pre 1.60 ml, 95%CI 1.51.-1.69 vs. mean post 1.55, 95%CI 1.46–1.63; *p* = 0.0022) and also in the parahippocampal gyri volumes (mean pre 2.00 ml, 95%CI 1.84–2.16 vs. mean post 1.95, 95%CI 1.79–2.11; *p* = 0.0008). There was, however, no significant total volumetric change in the hippocampus (mean pre 4.04 ml, 95%CI 3.84–4.24 vs. mean post 4.08, 95%CI 3.89–4.26; *p* = 1.688; Fig. 6). Overall, we found no significant difference between pre- or post-interventional volumes and matched normal controls.

## Discussion

Our comprehensive analysis of cerebral microstructure revealed changes in the distribution of intracerebral free water in SIH patients, which seem to be modified by closure of the underlying spinal CSF leak. In summary, after leak closure, there was an increase in interstitial free water on a global gray matter level, but also involving the corona radiata, as well as the hippocampus and the parahippocampal gyri.

Interstitial fluid as a component of the glymphatic model is driven by arterial pulsations through the brain parenchyma and the exchange of CSF with interstitial fluid facilitated by Aquaporin-4 (AQP4) channels on astrocytic endfeet [39]. Efflux follows perivenulous channels into larger veins, returning solutes into the systemic circulation [39]. The function of this system is based on a complex interaction being influenced by arteriovenous pressure gradients and integrity of perivascular spaces [40]. Longitudinal MRI scans after intrathecal injections of Gadolinium-based contrast agents have shown a reduced tracer uptake of the brain parenchyma in SIH patients indicating that CSF pressure may be another relevant factor for glymphatic flow [17]. It is therefore reasonable that spinal CSF leaks lead to decreased glymphatic flow and a reduction in free interstitial water. Further mechanisms that have been proposed to explain altered glymphatics in SIH include narrowing or collapse of perivascular spaces, alterations in venous compliance and altered astrocytic function or AQP4-expression [40]. However, it should be noted that the physiological mechanisms of the glymphatic system in humans with regard to CSF pressure and cerebrospinal fluid loss diseases remain unclear.

In our analysis, gray matter structures, particularly the hippocampi tended to be more affected than white matter structures. Previous studies demonstrated a link between hippocampal volume and memory decline in patients with neurodegenerative disease [41, 42]. One investigation on microstructural changes in Alzheimer’s disease revealed increasing interstitial fluid, which likely reflects a volume expansion inversely correlated to the neuronal loss in the course of the disease [43]. In contrast to irreversible volumetric changes in the course of age-related neurodegenerative diseases, the reversible alterations observed in our cohort may reflect transient changes resolving by restoration of CSF homeostasis [14]. Due to differences in the vascularization compared to other brain regions, perfusion and thus the function of the hippocampus is very sensitive to compartmental volumetric changes [44, 45]. Given the high metabolic activity of the hippocampus, this region is thought to be particularly reliant on efficient glymphatic flow to maintain homeostasis [44]. The relationship between increased glymphatic activity and gyrification has already been demonstrated in different animal species [46]. Although little is known about the different kinetics of gray and white matter glymphatic flow in humans, a reduction in CSF pressure could particularly affect areas with a higher metabolic activity or a higher turnover of interstitial water fluid.

Studies on macroscopic volume shifts in SIH demonstrated increasing ventricular volume after closure of the CSF leak [3, 4]. Our study complements those data with observations on interstitial fluid in different cerebral regions. Despite detecting no significant change in V-CSF in the white matter on a global level, there was a significant increase within the corona radiata. In comparison, in patients with idiopathic normal pressure hydrocephalus, periventricular white matter signal alterations were found to have higher free water fractions than those with microangiopathic lesions, which also correlated with an increased ventricular volume [7]. In the context of a spinal CSF leak, we found a consistent effect of low ventricular volumes and decreased free interstitial fluid.

We detected a significant V-CSF decrease in the posterior but not in the anterior and superior corona radiata. This difference likely reflects a spatial asymmetry of brain shift and interstitial fluid redistribution in SIH patients. Voxel-based approaches showed that volumetric brain shift in SIH patients probably does not occur symmetrically in three dimensions, but an anterior-to-posterior gradient is present [4]. Furthermore, orthostatic headache - a hallmark symptom of SIH - often compels patients to remain predominantly supine for weeks or even months. A recent study identified body position as an influencing factor on the glymphatic system in SIH, with decreased glymphatic influx in standing position [47]. Furthermore, current research on phase contrast MRI showed increased craniocervical CSF flow in supine vs. upright position [48], probably promoting CSF exchange and glymphatic clearance in a supine position. The extent to which body posture and/or the spatial asymmetry of the brain shift in SIH patients leads to an altered water distribution in the brain remains unclear. Nevertheless, our results indicate a spatial asymmetry of interstitial free water redistribution and provide a possible methodological approach for future studies.

The question also arises as to why only partial and only minor volumetric changes can be detected in the compartments under consideration. One can assume that on a global level, the microstructural changes between the compartments are “balanced”, i.e. possibly water shifts between glial cells/neurons and interstitium take place, so that on a macroscopic level no change in gross volume is detectable. Nevertheless, we noted a decrease in both the amygdala as well as parahippocampal gyri volume post-treatment. Such a volumetric recompensation is conceivable in the case of a previously reduced subarachnoid space or enlarged venous compartments. Another explanation might be a decline in brain sagging and therefore decreasing downward pressure on these regions. To understand these mechanisms, we need more comprehensive volumetric data of the microvascular as well as the subarachnoid compartment, which should be the goal of future research.

Our findings indicate changes in the cerebral free water distribution on a microscopic level, which needs correlation with clinical functional data including neuropsychological testing with region-specific assessments. As shown in a prior study, cognitive deficits are seen in a proportion of SIH patients [14]. Here, SIH patients rather presented with attention and executive than episodic memory deficits [14]. In a subset of patients in our cohort, MoCA Scores were available, indicating increased scores after therapy. However, these data do not allow for firm conclusions and warrant further investigation in a larger sample size.

Our study investigated a limited sample size, while including high quality imaging data and advanced postprocessing, which is more difficult to acquire in larger numbers of patients. We further included patients with different leak types and also different methods of leak closure.

One particular strength of our approach is the use of individual FreeSurfer and not SPM-12 based segmentations, to minimize the effects of brain shift. However, small partial volume effects cannot be completely eliminated.

## Conclusion

We analyzed multi-shell diffusion MRI data in patients with SIH focusing on the interstitial fluid of a three-compartment model. Compared to normal controls, spinal CSF loss is associated with a decrease in free water in the hippocampi and posterior corona radiata. Closure of a spinal CSF leak leads to a decent, but significant increase of free water in the gray matter, corona radiata, hippocampi, and parahippocampal gyri. The observed shifts in brain interstitial fluid affecting key cerebral regions, such as the hippocampi, offer valuable insights into the potential mechanisms contributing to cognitive decline in patients with CSF loss, which needs further investigation including comprehensive neuropsychological data.

## Data Availability

The datasets used and analysed during the current study are available from the corresponding author on reasonable request.

## Abbreviations

CVF: CSF venous fistula
CTM: CT-myelography
DMI: Diffusion microstructure Imaging
DSM: Digital subtraction myelography
NC: Normal controls
SIH: Spontaneous intracranial hypotension
